# Leaf functional diversity and environmental filtering in a tropical dry forest: Comparison between two geological substrates

**DOI:** 10.1002/ece3.10491

**Published:** 2023-09-05

**Authors:** Valentina Sandoval‐Granillo, Jorge A. Meave

**Affiliations:** ^1^ Departamento de Ecología y Recursos Naturales, Facultad de Ciencias Universidad Nacional Autónoma de México Ciudad de México Mexico

**Keywords:** intraspecific functional variation, leaf dry matter content, leaf functional traits, leaf thickness, soil properties, specific leaf area

## Abstract

The role of geological substrate in shaping plant community functional diversity remains poorly understood. Considering the involvement of leaves in the energy, water, and nutrient economics of plants, we hypothesized that leaves experience geology‐related filtering, which in turn shapes their functional attributes and community leaf functional diversity on different substrates. We studied tropical dry forest communities on limestone and siliciclastic phyllite‐derived soils, comparing their functional diversity and soil physico‐chemical properties. We predicted the most benign habitat (less severe filter) to be associated with higher leaf functional diversity and an acquisitive strategy prevalence, while the more stressful habitat should show conservative leaf traits and lower leaf functional diversity. We measured six traits in 31 common tree species (representing ~80% of community crown cover): leaf area, specific leaf area, leaf thickness, leaf dry matter content, petiole length, and leaf blade narrowness. Leaf functional diversity was assessed through the functional trait dispersion metric. Intraspecific functional variation was examined in 25 species shared between substrates. The limestone substrate was more fertile (higher phosphorous) with higher water retention, while phyllite had higher nitrogen and lower humidity. Principal component analysis segregated plots by substrate, with limestone plots being more clustered. Community leaf functional diversity was higher in the limestone forest. Most species examined showed inter‐substrate trait differences in at least one leaf functional trait. The two substrates constituted distinct growth environments, with the more benign substrate associated with higher community leaf functional diversity. The intraspecific analysis revealed the prevalence of acquisitive traits in the more benign and more conservative traits in the more stressful habitat. This study advances our understanding of the role of geological substrate as an environmental filter in tropical dry forests, influencing leaf functional responses and emphasizing the importance of intraspecific functional variation.

## Graphical abstract

This study sheds light on how lithological substrates can shape community leaf functional diversity by creating environmental filters of varying intensities. It highlights the importance of studying intraspecific functional variation and provides an understanding of the complex link between soil and leaf functional traits.

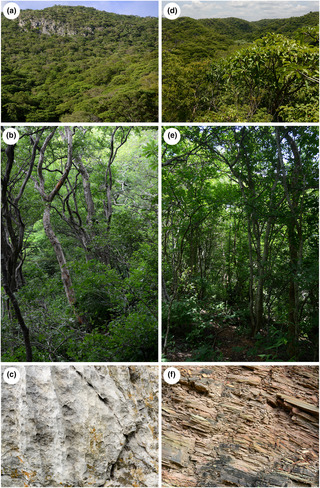

## 1 INTRODUCTION

Leaves are the fundamental photosynthetic units of plants and the regulatory organs of transpiration and carbon assimilation (Govindjee & Whitmarsh, 1982; Gurevitch et al., 2006). They are continuously subjected to a wide range of selective pressures, which has resulted in extraordinary morphological, physiological, and anatomical diversification (Givnish, 1987). Hence, leaves are highly sensitive environmental sensors that provide plants with valuable information about their habitats (Givnish, 1984; Roa‐Fuentes et al., 2015; Yun & Taylor, 1986). Most leaf characteristics are essential to their own functioning and that of the entire plant, and thus are considered functional traits (Díaz et al., 2004; Poorter & Bongers, 2006; Reich et al., 1999), as they are morpho‐physio‐phenological characteristics that indirectly impact fitness through their effects on growth, reproduction, and survival (Violle et al., 2007). For example, leaf area, leaf thickness, specific leaf area (SLA), and leaf dry matter content (LDMC) are basic functional traits related to nutrient acquisition and use (Table 1; Pérez‐Harguindeguy et al., 2013; Reich et al., 1997), and thus are essential components of the leaf economic spectrum (Wright et al., 2004). Functional attributes are the values or states that a functional trait can take (Violle et al., 2007); the range and distribution of functional trait values (i.e., attributes) in a community were defined by Lavorel et al. (2008) as the functional diversity of the community.

**TABLE 1:** Four of the most representative leaf traits along with their calculations and ecological interpretations.

Functional trait	Calculation	Ecological interpretation
Leaf area	Direct measurement	Directly related to light capture and hydric balance. Leaf area diminishes under water stress, extreme temperatures, and high radiation
Specific leaf area (SLA)	$\frac{\text{Fresh leaf area}\;\left({\mathrm{mm}}^{2}\right)}{\text{Dry}\;\text{mass}\;\left(g\right)}$	Related to resource capture and use in plants. It rises under conditions of high soil water and nutrient availability. Responds negatively to photon irradiance
Leaf thickness	Direct measurement	Positively related to leaf hydric efficiency and longevity, it rises in arid climates
Leaf dry matter content (LDMC)	$\frac{\text{Dry}\;\text{mass}\;\left(\mathrm{mg}\right)}{\text{Fresh weight}\;\left(g\right)}$	Negatively correlated to SLA and relative growth rate. Higher LDMC gives leaves a higher longevity and resistance. It rises under low water and nutrient contents in soil

Biogeographic and environmental factors, dispersal mechanisms, and internal community dynamics are the three levels of filtering involved in community assembly processes (Lortie et al., 2004). These filters can only be overcome by species possessing particular functional attributes that increase their chances of establishing and surviving in a particular habitat (Bazzaz, 1991). The environmental filtering hypothesis predicts that species with similar attributes are selected by the abiotic environment in order to become members of the community (Grime, 2006; Keddy, 1992; Le Bagousse‐Pinguet et al., 2017; Weiher et al., 1998). For example, in highly stressful habitats species that are less tolerant to the particular stress operating there will be filtered out of the community, thus reducing the range of attributes occurring in it (Cornwell & Ackerly, 2009). Conversely, more benign conditions, i.e., where limiting conditions do not strongly impact individual performance (such as milder temperatures, deeper and richer soils, and higher water availability) have been associated with more functionally diverse plant communities (Ding et al., 2019; Ottaviani et al., 2016; Spasojevic et al., 2013). The lithology and morphogenetic characteristics of geological substrates have effects on topography, soil nutrient availability, and soil physicochemical properties, all of which induce changes in species composition and abundance (de Souza et al., 2023; Durán et al., 2006; Gerrard, 1992; Muñoz et al., 2023; Pérez‐García et al., 2010). Geological substrate, a relevant factor for plant growth, can act as an environmental filter capable of shaping species distributions and functional diversity, depending on variations in the water retention potential and fertility of its derived soil (Fayolle et al., 2012). Nutrient‐based species selection is an important driver of community assembly, and community functional space size increases with soil nutrient concentrations (Peguero et al., 2023). Locally, substrate type and changes in elevation that affect soil depth, nutrient availability, and soil texture result in contrasting plant functional strategies (de Souza et al., 2023).

**FIGURE 1:**
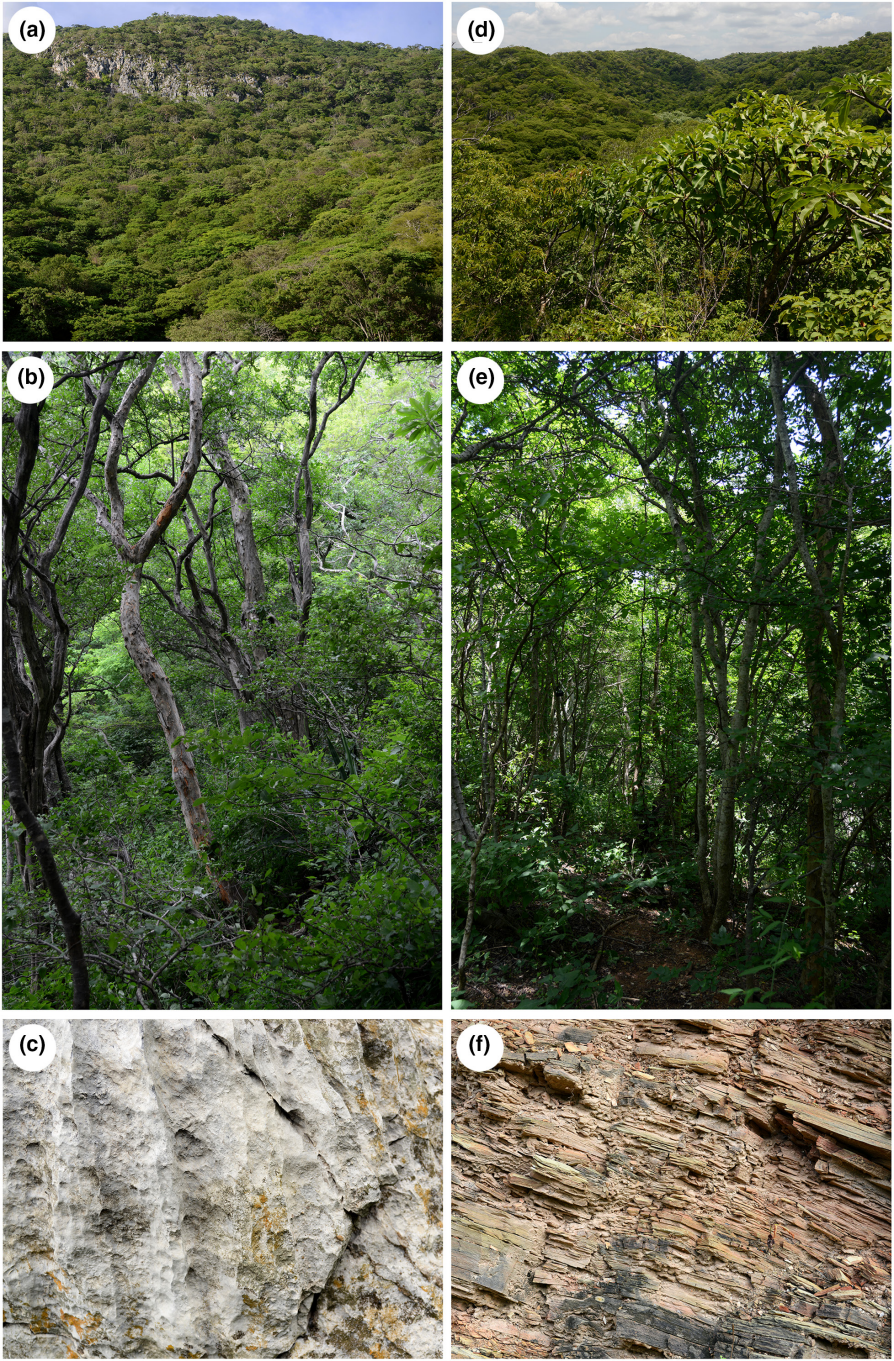
Images of the tropical dry forest communities in Nizanda, Oaxaca, southern Mexico, where this study was conducted. Tropical dry forest developing on limestone substrate: (a) external view in the wet season, (b) forest interior, (c) limestone parental rock. Tropical dry forest developing on siliciclastic phyllite: (d) external view in the wet season, (e) forest interior, (f) siliciclastic phyllite parental rock. Photographs: César Miguel‐Talonia.

Half of the global tropics have a seasonally dry climate in which tropical dry forests or savannahs occur (Pennington et al., 2018). Tropical dry forests are biomes with strong seasonal water limitations associated with a deciduous phenology during the dry season; their soils, usually richer and less acidic than savannah soils (Pennington et al., 2018), allow the development of closed canopies that inhibit grassy growth and fire occurrence (Murphy & Lugo, 1986; Pennington et al., 2000). In the region of Nizanda (S Mexico), two different tropical dry forest communities occur, each one being associated with a different geological substrate (limestone or siliciclastic phyllite; Figure 1). Previous work in this region has shown that despite shared characteristics that warrant their classification as the same vegetation type (Pérez‐García et al., 2010), notable differences exist between them: the forest associated with limestone is more developed (e.g., it has higher basal area and density, and higher diversity), suggesting that it grows in a more benign habitat than its counterpart growing on phyllite (Muñoz et al., 2023). These contrasts motivated us to compare the functional diversity of these two tropical dry forest communities to test the hypothesis that geological substrate constitutes an environmental filter that shapes community leaf functional diversity. To this end, we first compare a set of edaphic variables between the two substrates and then we characterize and compare the leaf functional diversity by calculating functional diversity metrics. As most species are shared by both communities, we also examined intraspecific differences in their functional attributes.

In line with the hypothesized substrate‐related differential filtering, we predicted (1) the existence of a different leaf functional diversity for each of these tropical dry forest communities, (2) higher functional diversity in the forest growing on limestone if the level of environmental restriction is related to a broader leaf functional range, and (3) an association of the limestone forest with more acquisitive traits (i.e. higher leaf area and SLA) and of the phyllite forest with more conservative traits (i.e. higher LDMC and leaf thickness). We tested these predictions by examining leaf functional diversity in each forest type in the calculation of the functional trait dispersion metric and its components, as defined by Scheiner et al. (2017).

## 2 MATERIALS AND METHODS

### 2.1 Study site

The study was conducted in Nizanda (16°39′30″ N, 95°00′40″ W), Oaxaca state, southern Mexico. The regional climate is Aw or equatorial savannah with dry winter (Kottek et al., 2006) with annual precipitation of 902.6 mm on average and a mean annual temperature of 27.6°C (CLICOM, 2020). Seven vegetation types have been reported around Nizanda, among which savannah and tropical dry forest (TDF) are the most extensive ones (Pérez‐García et al., 2010). In the region, two main geological substrates underly the TDF: siliciclastic phyllite and limestone (Pérez‐Gutiérrez et al., 2009). The siliciclastic phyllite is stratified in layers 0.2–10 cm thick, with fine to medium grano‐lepidoblastic texture, and is composed of minerals with a tabular habit (chlorite and muscovite), graphite, quartz inclusions, and feldspar and iron oxide subrounded grains parallelly disposed to the schistosity surface. The limestone rock of Albian‐Cenomanian age is stratified in layers 50–80 cm thick. Limestone rides on top of the siliciclastic phyllite.

### 2.2 Soil sampling, processing, and analysis

Soil samples were collected in 14 permanent plots of old‐growth TDF, seven located on the limestone‐derived substrate (henceforth ‘limestone forest’) and seven on the phyllite‐derived substrate (henceforth ‘phyllite forest’; Table 2). For density, water content, and porosity measurements, five samples were taken from homogenously distributed points in each plot using a 100‐cm^3^ stainless steel cylinder, and individually stored in plastic bags. Water content was determined directly with the aid of the gravimetric method and particle density was determined using the pycnometer method. For all other measurements, a compound 1 kg sample was taken from each plot, mixing additional soil from the same five points (for detailed soil analysis protocols see Supplementary Material).

**TABLE 2:** Scale of inference and replication level of the study.

Scale of inference	Scale at which the factor of interest is applied	Number of replicates at the appropriate scale
Substrate type (soils)	Plots	7 combined samples per substrate
Community type (functional profiles)	Plots	Data combined from 7 plots in phyllite and 7 plots in limestone
Species	Individuals	5 individuals of each species per substrate

*Note*: Lithological substrate type has two levels (phyllite‐derived substrate and limestone‐derived substrate). Soil samples were collected, and leaf measurements were done on trees located in seven plots (500 m^2^) belonging to each substrate (i.e. 3500 m^2^ per substrate). Community type (by substrate) was the unit of analysis for functional indices calculation, which required combining the data from the seven study plots for each substrate. The leaves of five individuals per species of the 25 species shared between substrates were measured in each substrate type.

Soil samples were oven‐dried at 105°C for 12 h for soil bulk and particle density determinations. Soil water content was determined gravimetrically, and soil pH and conductivity were measured in the compound soil samples. Na and K contents were determined by flame emission spectroscopy, and Ca and Mg by atom absorption spectroscopy using standard routines; available phosphorous content was determined by the Bray and Kurtz (1945) method using a spectrophotometer, total nitrate and ammonia contents were measured by potassium chloride extraction with the modified method suggested by Strickland and Parsons (1972), and soil organic matter content was determined through calcination. Soil texture was determined using a modification of the Bouyoucos (1962). Soil analyses were conducted in the Environmental Analysis Unit, Faculty of Sciences, Universidad Nacional Autónoma de México.

A principal component analysis was conducted using the normalized and standardized quantitative soil variables. Due to PCA sensitivity to redundant variables (Ivosev et al., 2008), soil ammonia, nitrate and silt contents, and particle density were excluded from this analysis. Next, the characteristics of the soils derived from the two substrates were compared through linear modeling; for each soil variable, a null model and a model including the plot's substrate as a fixed factor were constructed (both models included the plot as a random effect). The sample corrected Akaike Information Criterion (AICc) was calculated for each model, along with ΔAICc (i.e., the differences between each AICc and the smallest AICc) and Akaike weights. If ΔAICc <2, both models were considered to be equally supported (Burnham & Anderson, 2002).

### 2.3 Leaf sampling and functional trait calculations

Thirty‐two species with the highest cumulative crown cover and biomass were selected from a database containing tree‐by‐tree information from old‐growth forest plots in Nizanda, each 500 m^2^ in size. Leaf canopy cover was given higher priority as a criterion for species selection, as this structural variable best reflects the amount of foliage corresponding to the different leaf types. Among the selected species, 25 were shared between the two forests, four were exclusive to the limestone forest and three to the phyllite forest. In each forest type, five individuals were chosen for each species (i.e., 10 individuals for species occurring in both forest types), and five sun leaves were collected from each individual with the aid of a telescopic tree pruner (Table 2; Pérez‐Harguindeguy et al., 2013). Samples were wrapped in moist paper towels, placed in sealed bags with CO_2_ exhaled into them, and stored in a cooler. Approximately 5 h after sampling, the leaves were patted dry, scanned with 600‐dpi resolution (Scanjet G3110, Hewlett Packard), measured using the Image J software (Abràmoff et al., 2004), and weighed using an analytic scale (Adventurer N13123, Ohaus). Leaf thickness was measured with a digital micrometer (0.001 mm Digimatic 293‐831, Mitutoyo) halfway between the leaf base and its apex, avoiding primary and secondary venations; for compound leaves, three folioles were measured and the average measurement between them was recorded. As foliolules were too fragile to be measured individually, their leaf thickness was calculated as follows (Vile et al., 2005):

$${\left(\mathrm{SLA}\times \text{LDMC}\right)}^{-1}.$$

Leaves were oven‐dried at 70°C for 72 h for leaf dry mass determination; SLA, LDMC, and leaf narrowness were calculated using standard formulae (Pérez‐Harguindeguy et al., 2013). Calculations of SLA and LDMC were made in three different ways: (1) leaf blade including the petiole, (2) leaf blade without petiole, (3) minimum photosynthetic unit (MPU; i.e., the foliole or the foliolule) including the petiole or the petiolule; this procedure was aimed to compare the results of the three calculations, as standardized protocols are inconclusive regarding the inclusion of the petiole and the use of the MPU instead of the leaf blade in species with compound leaves (Cornelissen et al., 2003; Garnier et al., 2001; Pérez‐Harguindeguy et al., 2013), which are common in TDF.

Community functional diversity was first examined through frequency distribution histograms for each functional trait by substrate type, which we refer to as leaf functional profiles for brevity. Additionally, community‐weighted means (CWM) for traits in each forest type were calculated by multiplying trait values for the species in each site by their relative canopy cover in that site, adding these values, and then dividing by the sum of canopy cover values (Lavorel et al., 2008).

### 2.4 Leaf functional analysis

The leaf functional analysis included two steps, one concerning the community level and involving calculations of functional diversity indices and functional diversity profiles (frequency distributions of leaf trait values), and another concerning intraspecific variation analyses.

Between‐substrate comparison of functional diversity was based on a functional trait dispersion metric that integrates three diversity components: species richness, mean dispersion (of functional traits), and evenness (Scheiner et al., 2017); thanks to this feature, it can be decomposed to understand patterns of variation between communities, as a greater integrative metric in one community does not necessarily mean greater evenness, dispersion, and richness compared to another. The functional trait dispersion metric and its components are based on the Hill function (Hill, 1973), which is expressed as:

$${}^{q}\;D\left(A\right)=\sum_{i=1}^{S}{\left(p_{i}^{q}\right)}^{1/\left(1-q\right)}$$

where *A* indicates diversity based on abundance, *p*_*i*_ is the proportional abundance of the *i*th species, and *q* determines the weighting of proportional abundances.

The first step to calculate the functional trait dispersion metric is the measurement of the variability among the between‐species pairwise distances (${}^{q}\;H\left(T\right)$), which is achieved with the Hill function:

$${}^{q}\;H\left(T\right)={\left(\sum_{i}^{S}\sum_{j}^{S}f_{\mathrm{ij}}^{q}\right)}^{1/\left(1-q\right)}$$

where *S* is the number of species and *f*_*ij*_ indicates the proportional distance between the *i*th and *j*th species. Next, mean dispersion of the functional traits (*M*) is calculated as:

$$M=\sum_{i}^{S}\sum_{j}^{S}d_{\mathrm{ij}}/S^{2}$$

where *d*_*ij*_ represents the standardized pairwise distance in trait space; *d*_*ij*_ is calculated as the Manhattan distance.

Then, functional‐trait species diversity (^*q*^ *D*(*T*)) is calculated as:

$${}^{q}\;D\left(T\right)=\frac{1+\sqrt{1+4{}^{q}\;H\left(T\right)}}{2}.$$

Functional evenness, that is, the extent to which species are equally distributed in functional space (^*q*^ *E*(*T*)) can be described as:

$${}^{q}\;E\left(T\right)={}^{q}\;D\left(T\right)/S.$$

At last, the functional trait dispersion metric is a multiplicative relation, as follows:

$${}^{q}\;D\left(\mathrm{TM}\right)=1+{}^{q}\;D\left(T\right)\times M.$$

The value derived from this metric (the “effective number” of species) represents the proportional reduction of species richness in the community due to deviations from a perfectly equitable dispersion of species. It evaluates the number of functionally distinct species according to the among‐species distance in the multidimensional space defined by trait values. A *q* value of 1 was used to count species in proportion to their relative canopy cover. *M′*, or mean dispersion was used instead of *M*, where:

$$M^{\prime }=\frac{S}{S-1}M$$

*M′* has the range [0, 1] and is calculated as the average distance among all possible pairs of species. As such, functional trait dispersion can also be decomposed as:

$${}^{q}\;D\left(\mathrm{TM}\right)=1+\left(S-1\right)\times {}^{q}\;E\left(T\right)\times M^{\prime }.$$

Lithology‐related intraspecific variation in those species shared between communities was analyzed with a Mann–Whitney *U*‐test (*α* = .05). Species that differed significantly were plotted, along with species differing with marginal significance (.05 < *p* < .07). Due to the uneven occurrence of species in the study plots, substrate type was the unit of analysis for functional indices calculation, which required combining the data from the seven study plots for each substrate.

## 3 RESULTS

### 3.1 Geology contrasts in soil properties

Five of the 15 edaphic variables analyzed (pH, electrical conductivity, calcium, available phosphorous, and porosity) had higher means in the limestone than in the phyllite substrate (Table A1 in Appendix 1). By contrast, mean nitrates, ammonium, and total nitrogen values were higher in the phyllite. For these eight variables, the best model included substrate as a factor (Table 3). Conversely, the null model was a better fit for sodium concentration, bulk density, and particle density. In the case of organic matter content, gravimetric humidity, and magnesium and potassium concentrations, neither model had a good fit (Table 3).

**TABLE 3:** Comparison between the null models and models including substrate type as a factor explaining edaphic heterogeneity between limestone and phyllite‐derived substrates.

Edaphic variable	Competing models	AICc	ΔAICc	AICc Wt
pH	Null	−3.5	6.93	0.03
	Substrate*	−10.44	0	0.97
Conductivity	Null	323.01	9.37	0.01
	Substrate*	313.64	0	0.99
Calcium	Null	223.38	5.86	0.05
	Substrate*	217.51	0	0.95
Magnesium	Null	120.32	0	0.64
	Substrate	121.48	1.16	0.36
Sodium	Null*	−54.51	0	0.98
	Substrate	−46.9	7.61	0.02
Potassium	Null	−28.72	0	0.55
	Substrate	−28.32	0.39	0.45
Available phosphorous	Null	163.93	4.04	0.12
	Substrate*	159.89	0	0.88
Total nitrogen	Null	257.09	6.33	0.04
	Substrate*	250.75	0	0.96
Nitrates	Null	255.39	5.88	0.05
	Substrate*	249.51	0	0.95
Ammonium	Null	150.15	2.52	0.22
	Substrate*	147.63	0	0.78
Organic matter	Null	149.14	1.81	0.29
	Substrate	147.34	0	0.71
Particle density	Null*	−8.21	0	0.88
	Substrate	−4.15	4.06	0.12
Bulk density	Null*	−41.0	0	0.94
	Substrate	−35.4	5.6	0.06
Porosity	Null	461.53	3.28	0.16
	Substrate*	458.25	0	0.84
Gravimetric humidity	Null	255.29	1.99	0.27
	Substrate	253.31	0	0.73

*Note*: The asterisk (*) indicates the best model for each variable (i.e. ΔAICc >2.0).

Abbreviations: AICc Wt, model weight; AICc, sample‐corrected Akaike Information Criterion; ΔAICc, difference between AICc values for both models compared for each variable.

Coefficients of variation were considerably larger for Mg, K, P, nitrates, ammonium, and the silt and sand fractions in the limestone, unlike pH, total N, bulk density, porosity, and clay fraction, which were more variable in phyllite plots. In turn, the six remaining variables had similar coefficients of variation in both substrates.

**FIGURE 2:**
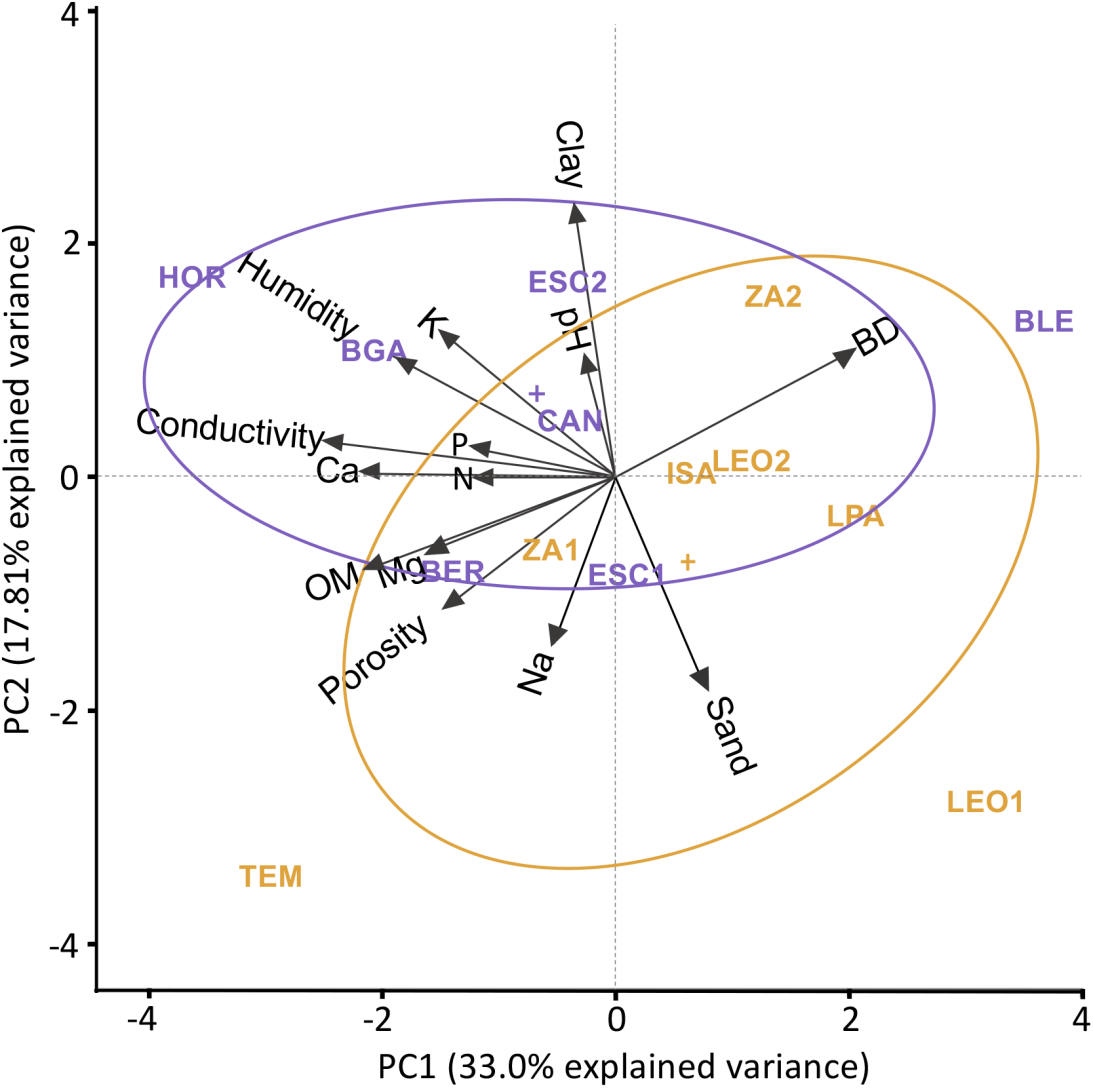
Principal component analysis (PCA) of the study sites based on a matrix of standardized edaphic variables. The percent variance explained by the first two components was 50.9%. Limestone plots (purple): HOR, BGA, ESC2, CAN, BER, ESC1, BLE. Phyllite plots (yellow): TEM, ZA1, ISA, LEO2, ZA2, LPA, LEO1. Edaphic variables abbreviations: BD, bulk density; Ca, calcium; Humidity, Gravimetric humidity; K, potassium; N, nitrogen; Na, sodium; OM, organic matter; P, available phosphorous. The ellipses represent the mean distance to the centroid of each substrate; centroids are indicated with a cross.

The first two PCA components together explained a little over half of the data's total variance (50.9%; Figure 2). Five variables showed strong orthogonality. Ca, N, P, and conductivity were associated with PC1 only, while pH and the clay and sand fractions were more strongly associated with PC2. The PCA produced a clear segregation of the plots by substrate along PC1 and also along PC2, albeit less evidently, which resulted in an oblique spatial distribution. Remarkably, substrate‐related plot aggregation was not perfect since some plots were placed far from the rest of the plots that shared substrate (e.g., BLE in limestone and TEM in phyllite). More importantly, limestone plots were more compactly aggregated compared to the phyllite plots, indicating a higher between‐plot similarity on this substrate. Most edaphic vectors were negatively associated with PC1, suggesting that this is an axis of fertility, with the highest values coinciding with the location of limestone plots on the ordination space.

### 3.2 Plant community functional profiles and functional diversity on the two substrates

**FIGURE 3:**
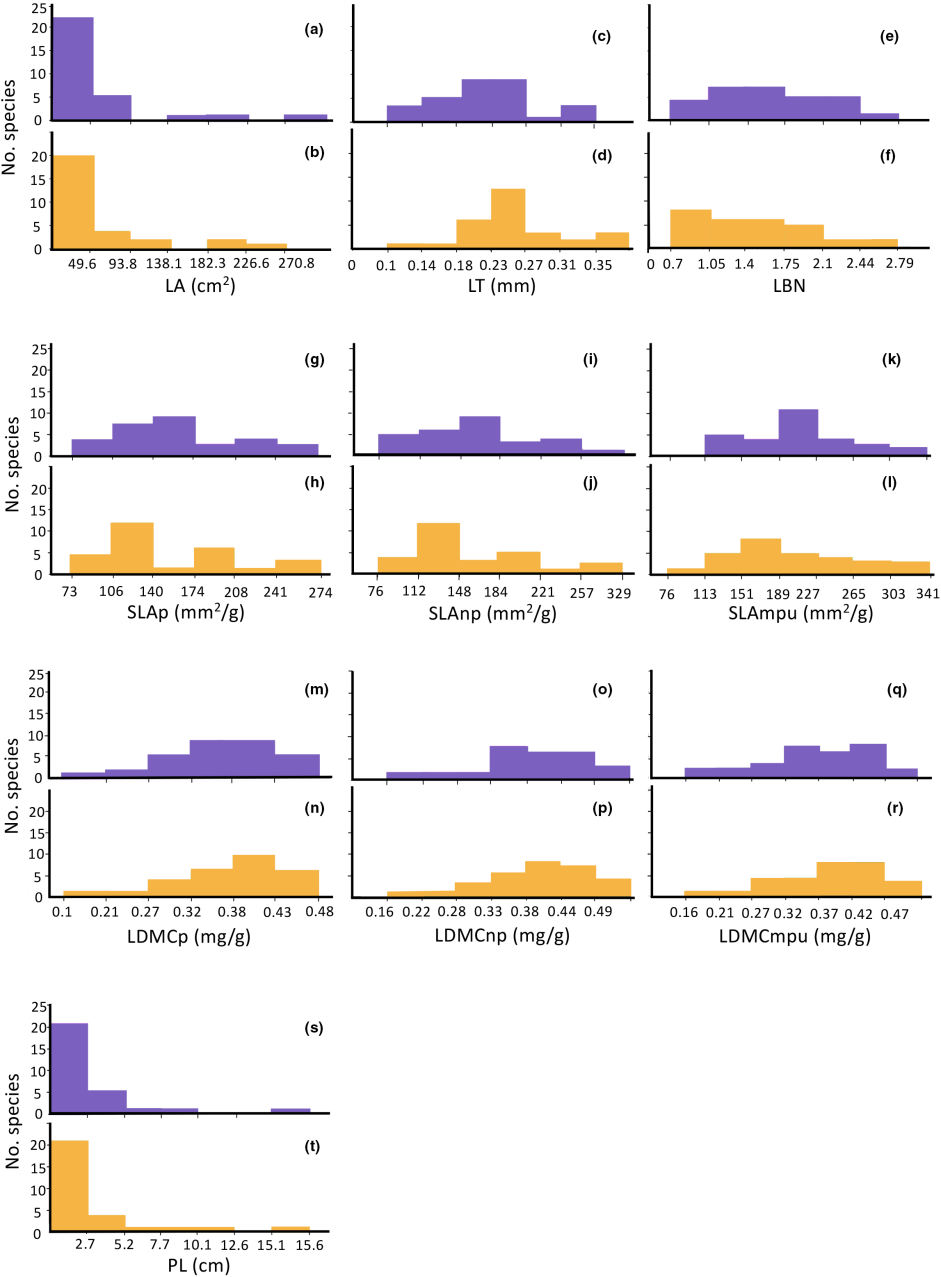
Community functional profiles for 10 functional traits in limestone (purple) and phyllite (yellow) forests. Functional trait abbreviations: LA, Leaf area (a, b); LT, Leaf thickness (c, d); LBN, leaf blade narrowness (e, f); SLAp, SLA with petiole (g, h); SLAnp, SLA without petiole (i, j); SLAmpu, SLA with the minimum photosynthetic unit (k, l); LDMCp, LDMC with petiole (m, n); LDMCnp, LDMC without petiole (o, p); LDMCmpu, LDMC with the minimum photosynthetic unit (q, r); PL, petiole length (s, t).

The functional profiles for each trait were similar between substrates but some point differences stand out (Figure 3). First, the leaf area profile for the limestone forest had a wider range (Figure 3a,b). Most other functional profiles were spread over the same range, except for leaf thickness (Figure 3c,d) and SLA calculated with the minimum photosynthetic unit (Figure 3k,l). The functional profiles for SLA and LDMC showed a more homogeneous species distribution in the limestone forest, while profiles for the phyllite forest tended to have many species concentrated in fewer classes, or several classes represented by a single species.

Community weighted means (CWMs) were lower than the simple means for leaf area, leaf blade narrowness, and petiole length on both substrates and for leaf thickness in the phyllite (Table 4); additionally, CWMs were higher than the simple means for all the SLA and LDMC calculations in both substrates along with leaf thickness in the limestone.

**TABLE 4:** Comparison between simple means and community weighted means (CMW) for 10 leaf functional traits in tropical dry forests on limestone and siliciclastic phyllite.

Trait	Limestone			Phyllite		
	Simple mean (SD)	CWM (SD)	Ratio	Simple mean (SD)	CWM (SD)	Ratio
LA	45.49 (59.8)	40.68 (49.3)	1.12	51.80 (60.44)	47.21 (59.46)	1.10
LT	0.19 (0.06)	0.19 (0.08)	0.97	0.19 (0.07)	0.18 (0.08)	1.04
SLAp	157.77 (49.43)	161.95 (65.27)	0.97	153.19 (55.43)	174.91 (73.98)	0.92
SLAnp	165.87 (54.04)	169.52 (70.22)	0.98	161.87 (60.36)	165.78 (80.12)	0.93
SLAmpu	173.52 (54.24)	180.79 (70.79)	0.96	171.66 (65.20)	189.12 (83.64)	0.91
LDMCp	0.35 (0.08)	0.36 (0.10)	0.97	0.36 (0.07)	0.38 (0.11)	0.94
LDMCnp	0.35 (0.08)	0.36 (0.10)	0.97	0.36 (0.07)	0.38 (0.10)	0.94
LDMCmpu	0.35 (0.08)	0.36 (0.10)	0.97	0.36 (0.07)	0.37 (0.10)	0.96
LBN	1.60 (0.51)	1.53 (0.60)	1.04	1.51 (0.57)	1.37 (0.59)	1.10
PL	2.38 (2.98)	2.06 (2.43)	1.15	2.72 (3.30)	2.62 (3.23)	1.04

Abbreviations: LA, leaf area; LDMCmpu, LDMC with minimum photosynthetic unit; LDMCnp, LDMC without petiole; LDMCp, LDMC with petiole; LT, leaf thickness; SLAmpu, SLA with minimum photosynthetic unit; SLAnp, SLA without petiole; SLAp, SLA with petiole.

**FIGURE 4:**
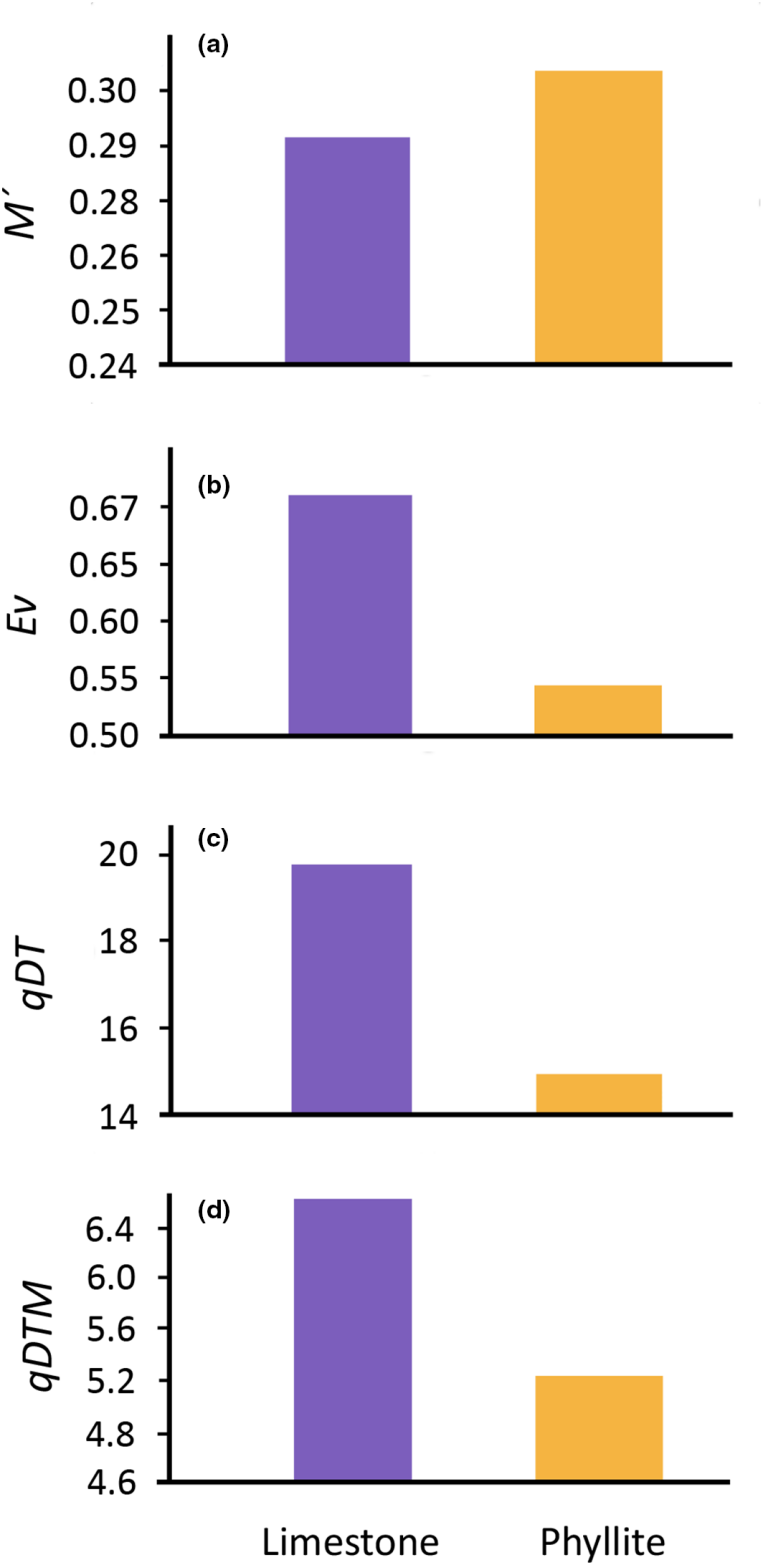
Comparison of functional diversity indices between substrates based on seven leaf functional traits measured in 29 common species occurring in the limestone forest (purple) and 28 common species in the phyllite forest (yellow). Indices based on SLA and LDMC were calculated with the leaf blade and the petiole. Functional diversity indices: *1DT*, effective number of equally distant species; *1DTM*, effective number of functionally distinct species; *Ev*, functional evenness; *M′*, standardized magnitude of dispersal of species' functional attributes. Note that the *Y*‐axes do not start at zero.

Figure 4 shows the functional diversity indices based on six leaf functional traits for the two forests studied. Only values calculated with both the leaf blade and petiole are shown since the three calculations of LDMC and SLA showed the same tendencies. The standardized attribute dispersion in functional space (*M′*) was larger in phyllite, unlike functional evenness (*Ev*), functional attribute diversity (*1DT*), and the effective number of functionally distinct species (*1DTM*), all of which were higher in limestone.

### 3.3 Intraspecific functional variation in species shared between substrates

**FIGURE 5:**
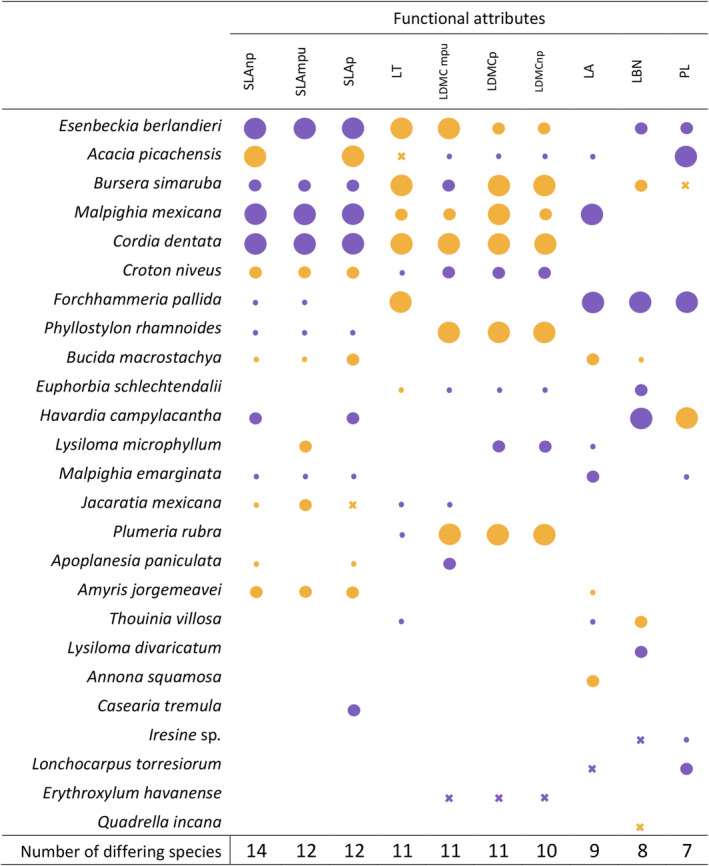
Leaf functional attribute comparison of the species shared between substrates, based on a Mann–Whitney *U*‐test. Traits: LA, leaf area; LBN, leaf blade narrowness; LDMCmpu, LDMC with the minimum photosynthetic unit; LDMCnp, LDMC without petiole; LDMCp, LDMC with petiole; LT, leaf thickness; PL, Petiole length; SLAmpu, SLA with minimum photosynthetic unit; SLAnp, SLA without petiole; SLAp, SLA with petiole. The circles represent attributes that differed significantly between substrates for each species (large circle, *p* < .001; medium circle, *p* < .01; small circle, *p* < .05; cross, *p* < .07). Color indicates the substrate having higher attributes: yellow, phyllite forest; purple, limestone forest. The total numbers of species whose attributes differed significantly between substrates are shown in the bottom row.

Figure 5 shows which functional traits were more strongly influenced by substrate type according to the number of species in which they differed. SLA was the trait most strongly influenced by substrate type, differing in 14 species when calculated without the petiole, and in 12 when calculated with the petiole and the minimum photosynthetic unit. Leaf thickness differed in 11 of the species as well as the LDMC calculated on the minimum photosynthetic unit, while LDMC with and without the petiole varied in 10 species. Leaf area differed in 9 of the shared species and leaf blade narrowness and petiole length differed in less than one‐third of the species shared between substrates. No functional trait differed significantly between substrates for all of the shared species.

The comparison of functional attributes between the 25 shared species showed an association of the intraspecific variation with substrate type (Figure 5). Species were distributed across all classes, and 13 out of the 25 species differed in at least half of the traits analyzed, 23 differed in at least one trait, and for *Quadrella incana* and *Erythroxylum havanense* LDMC and leaf blade narrowness differed with marginal significance, respectively. *Esenbeckia berlandieri* showed the highest sensitivity to substrate type, with 9 out of 10 traits differing between substrates. Most between‐substrate differences were significant, particularly for species differing in more than seven traits. Notably, for each trait a similar number of species had higher attributes either in limestone or in phyllite; however, more species had higher SLA in limestone, whereas the opposite pattern was found for LDMC (more species had higher attributes in the phyllite). The group of traits bound to light harvesting (leaf area, leaf petiole length, and leaf blade narrowness) showed a clear pattern of significantly higher attributes in the limestone forest, although they differed in only one‐third of the shared species.

## 4 DISCUSSION

We investigated whether the differences between limestone and phyllite‐derived substrates are strong enough to represent environments with differential environmental filtering, which could be reflected in different community leaf functional profiles. We showed that these substrates represent two growth environments differing in soil properties, which is not only reflected on the community‐level functional profiles but also at the intraspecific level. Below we further discuss these findings.

### 4.1 Do limestone and phyllite‐derived substrates represent different growth environments?

Nitrogen is the nutrient that plants require in the greatest quantities; in species with high photosynthetic capacities and a C3 metabolism, up to 75% of the nitrogen is invested in the photosynthetic apparatus (Chapin III et al., 1987). The interaction between water and nitrogen availability is central to plant function. In dry conditions, more water increases stomatal aperture and photosynthetic enzyme activity (Maroco et al., 2000). Due to its low concentration and solubility, phosphorous is also a critical nutrient limiting plant growth. It is essential for biochemical and physiological processes as an important component of membrane lipids, nucleic acids, and phosphorylated intermediaries of energy metabolism (Shen et al., 2011).

The differences in key nutrients (i.e., P, N) between phyllite and limestone‐derived soils confirm that these substrates offer two substantially different growth environments for plants. Yet, this differentiation is insufficient to sustain different vegetation types, despite the regional occurrence of various non‐TDF plant communities (Pérez‐García et al., 2010). The PCA suggests that the limestone substrate is more homogenous; moreover, the means of the edaphic variables along with the models used for the between‐substrate comparison suggest higher fertility for limestone (with higher Ca and P concentrations, higher electrical conductivity and porosity, and a pH closer to neutrality). The soils derived from both bedrocks are classified as non‐saline (< 0.7 dS/m, Ayers & Westcot, 1985). Electrical conductivity indicates soil salt content but also water content (Brevik et al., 2006; Mylavarapu et al., 1993). In turn, high soil porosity increases water and air movement, nutrient transport, root growth, and the presence of soil organisms. Zhalnina et al. (2014) reported a positive correlation between soil pH and plant richness with microbiota diversity and biomass. Notably, some soil variables were homogeneous between substrates; this was the case of soil organic matter content and gravimetric humidity, whose models did not reveal any substrate effects although both had higher mean values in the limestone substrate. Recent analyses have confirmed higher water contents for limestone‐derived soils (C. Miguel‐Talonia, unpublished) in accordance with our results. By contrast, the phyllite substrate stood out for having higher total nitrogen, ammonium, and nitrate concentrations, implying higher nitrogen availability in this environment. These differences aside, it is worth noting that the plot segregation on the PCA was not flawless; a likely explanation is that the limestone substrate rides on top of the phyllite in this geological terrain (Pérez‐Gutiérrez et al., 2009), which may cause the soil in some plots to be influenced by both parental rocks due to gravity‐driven movement of materials, preventing clear segregation. Despite these caveats, the evidence warrants the conclusion that each substrate represents a different growth environment for plants, potentially resulting in differential environmental filtering.

### 4.2 Do between‐substrate differences lead to community‐level functional differentiation?

The functional profile for the phyllite forest has a slightly larger dispersion in functional space (which increased when the petiole is included). Contrariwise, the functional profile for the limestone forest suggests a more equal species distribution, rendering it functionally more heterogeneous than the phyllite forest. Interestingly, CWMs for SLA and LDMC in both communities were higher than the simple trait means. This means that some of the most common species in both limestone and phyllite contribute to the CWM with large attributes for these traits. In turn, CWMs for traits directly linked with light capture were lower than simple trait means in both communities, i.e., the most common species in both substrates contribute to the CWM with small attributes for these traits. Leaf area and petiole length, traits that are related to light capture, were higher in the phyllite forest. Mean SLA was higher in limestone, while mean LDMC was higher in phyllite. However, the CWMs for both SLA and LDMC, two traits associated with resource acquisition (Cunningham et al., 1999; Vendramini et al., 2002; Wright et al., 2004), were higher in the phyllite forest except for the SLA calculation without the petiole. This is contrary to our predictions and an intriguing result, as a higher SLA indicates higher resource availability, whereas a higher LDMC is associated with environmental severity (Chaturvedi & Raghubanshi, 2013; Gotsch et al., 2010; Ordoñez et al., 2009; Tripathi et al., 2020), so that these traits are often negatively correlated (Pérez‐Harguindeguy et al., 2013). A possible explanation is a differentiated response between species of the phyllite forest, where some of the most common species respond to higher N concentrations with higher SLA, while others respond to a lower nutrient concentration and water availability with higher LDMC. Explanations for higher SLA in the phyllite substrate could be a positive correlation like the one reported by Ordoñez et al. (2009) between SLA and nitrogen mineralization, a proxy of N availability in the soil which would coincide with the soil results found for the phyllite. Moreover, Gong and Gao (2019) observed a strong correlation between pH and SLA, with the highest SLA found over a pH range of 5–6, which coincides with the pH recorded for this forest.

The functional indices calculated for the two communities being compared were consistent with the results of the functional profiles and the quantitative descriptions of community‐level traits. Functional diversity (*1DT*)—defined by the effective number of equally‐distant species—, functional evenness (*Ev*), and functional trait dispersion (*1DTM*), were higher in the limestone forest for the three SLA and LDMC calculations. Conversely, the standardized distance between leaf traits (*M´*) was slightly higher in the phyllite for the three calculations, with the calculation including the leaf blade and petiole showing the most noticeable differences between substrates, and the one based on the minimum photosynthetic unit being the least sensitive to these. Overall, values for functional trait dispersion (*1DTM*) were low (less than one‐fourth of total species richness), suggesting large functional redundancy in both substrates. This coincides with the findings of Scheiner et al. (2017) for a bat community in Peru; although their *1DT* values were closer to species richness, the *1DTM* decreased sharply. This result was interpreted as a high clustering of species with functionally similar responses.

The comparison between the two forest studied by us sheds new light on the particular roles of different environmental factors in defining the functional diversity of a given community. Peguero et al. (2023) found that functional space size and its filling increase with soil nutrient concentration, which is in agreement with our finding that functional space size is larger in the limestone forest, as this substrate is generally more fertile than the phyllite. Also, their more functionally diverse site had higher species richness and diversity, as is the case of our limestone forest. However, in their study, the fertility/functional space size association was linked to nitrogen, since the more functionally diverse site had higher nitrogen availability whilst both sites had similar phosphorous contents. Intriguingly, in Nizanda the least functionally diverse community (i.e., the phyllite forest) had higher soil nitrogen content, suggesting that the role of soil fertility may be overridden by water stress.

Our results support the main hypothesis, as between‐substrate differences were related to between‐community differences in the leaf functional profiles. Yet, the evidence for the second hypothesis (a strong relationship between the degree of environmental restriction and the leaf functional range) was weaker. According to this hypothesis, the limestone forest should have a more functionally diverse profile, and although this substrate type seems more benign, it is not so for all the edaphic variables analyzed. The phyllite substrate has higher nitrogen, a fundamental macronutrient for plant growth and development since it maximizes productivity, photosynthetic rate, and water use efficiency and absorption, providing adequate water availability (Nguyen et al., 2017). This is probably why the functional response was reverted with a higher *M′* in phyllite. Despite these caveats, the edaphic differences were undoubtedly linked with higher values of the functional indices in the limestone forest. Furthermore, the larger coefficients of variation for three essential nutrients (N, P, and K) in limestone suggest a higher spatial heterogeneity that allows more species (and their attributes) to enter the community, ultimately leading to higher functional diversity. These functional diversity indices align with the study of Cornwell and Ackerly (2009), which provided strong evidence for water availability‐mediated environmental filtering, resulting in a lower‐than‐expected functional attribute dispersion with low water availability. de Souza et al. (2023) reported an unexpected negative relation between two leaf traits, namely SLA and leaf nutrient concentrations, and soil nutrient availability; these authors attribute this finding to the fact that the nutrient‐rich site examined by them could have a lower soil water retention capacity due to reduced soil depth. Other studies have also reported high functional diversity in environments with more resources, deeper soils, higher water availability and more benign temperatures (Le Bagousse‐Pinguet et al., 2017; Ottaviani et al., 2016; Spasojevic et al., 2013). Ding et al. (2019) reported reductions in functional diversity indices, functional richness, SLA CWM, and leaf P and N concentrations along an altitudinal gradient over which both soil pH and mean annual temperature decreased. Regarding environmental filters imposed by climatic severity, TDFs have a higher functional diversity than expected compared to temperate seasonal forests, where functional attribute dispersion is lower than expected given their low species richness (Swenson et al., 2012, but see Šímová et al., 2015).

### 4.3 Is the functional response of species to the substrate homogenous?

Our results show that the between‐substrate edaphic differentiation affects the shared species between these communities in different ways. A remarkable finding is the continuum formed from species that are unaffected by the substrate to species that differed in nearly all functional traits examined. Apparently, species with similar responses differ regarding wood density, blade configuration, phylogeny or canopy height, but future work needs to formalize these relationships.

The leaf trait most strongly associated with substrate type was SLA which is a highly plastic functional trait that depends on the relation between foliar parenchyma and cell volume; it varies with light, nutrient and water availability, temperature, and soil compaction (Poorter et al., 2009; Shipley et al., 2016). Hence, it is not surprising that SLA was the most variable trait among species. Although the SLA CWM was higher in phyllite, implying higher SLA values for the most common species in this forest, the individual species analysis showed more limestone species with higher SLA. A likely explanation is that the leaves of plants growing on more water‐stressed substrates have lower expansion rates, and smaller and more compact cells with a lower fraction of air spaces (Maximov, 1929), resulting in lower SLA (Poorter et al., 2009). This possibility is consistent with the observation of lower SLA in phyllite, if the limestone is less water‐stressed. However, this would be viable only for trees with shallow roots that obtain water from the soil and not from deeper water deposits. Castellanos et al. (1991) reported that two‐thirds of the root biomass of shrubs, vines, and trees in the tropical dry forest of Chamela, Mexico, are found in the first 20 cm of the soil layer. Contrastingly, Freycon et al. (2015) reported that in semideciduous forests of the Central African Republic, 95% of the roots of *Entandrophragma cylindricum* (a common tree in this vegetation type) were found between depths of 258 and 564 cm.

Leaf thickness is negatively correlated with SLA, since SLA is a result of both density and thickness of the leaf tissue (Witkowski & Lamont, 1991). This relation is apparent in Figure 5, where species with significantly higher SLA in one substrate also had significantly higher leaf thickness in the other. Unfortunately, whether one substrate had higher leaf thickness than the other was unclear, since leaves have higher thickness in a similar number of species per substrate (see Figure 5).

Leaf dry matter content was also affected by substrate type, as it differed in nearly half of the shared species. This trait, in opposition to SLA, was generally higher in phyllite plots, in agreement with the CWM and the negative correlation between these two traits (Pérez‐Harguindeguy et al., 2013). Nonetheless, this result contrasts with the known negative relation between LDMC and soil N (Hodgson et al., 2011; Pinho et al., 2018), although species with higher LDMC in limestone cannot be ignored. These contrasting results show the difficulty of generalizing on the associations between leaf traits and the environment.

The three leaf traits bound to light capture were the least affected by substrate, differing in less than one‐third of shared species. Leaf area is tied to taxonomic identity more than SLA and it varies more between than within species (Campetella et al., 2019; Kichenin et al., 2013). Moreover, larger leaf blade narrowness and petiole length values contribute to a greater light capture per leaf area unit, by reducing foliar aggregation around stems (Niinemets et al., 2004; Takenaka, 1994). Higher petiole lengths reduce self‐shading, impacting the leaf blade angle (Niklas, 1991). Despite differing in a fewer number of species, these three traits showed a much more consistent tendency to be higher in the limestone forest compared to the other functional traits. Species responses to substrate type were heterogeneous. We observed a large range of response intensities. However, when traits were analyzed individually, SLA and LDMC clearly suggested a higher benignity for the limestone substrate.

Our species set comprised 32 common species in the two forests examined, which collectively account for ca. 80% of the total crown cover in each of these communities. Despite the small contributions to the forest canopy made by the excluded species which are rare in their respective forest, it is possible that the analysis of their leaf functional traits may be revealing regarding their subordinate condition. Their low abundance and crown cover might be precisely a result of their possession of attributes that are suboptimal to the environments in which they grow, affecting their fitness and therefore reducing their abundance, but not so inadequate as to cause their exclusion from these communities. This is an interesting question worth pursuing in future studies.

### 4.4 Methodological considerations

The three ways to calculate SLA and LDMC produced similar functional indices; this is relevant as measuring the area and weight of the minimum photosynthetic unit is considerably more time‐consuming. The most sensitive calculation to between‐substrate differences was the leaf blade without the petiole, which is the simplest measurement used here. This comparison is relevant because standardized manuals for the measurement of functional traits recommend the inclusion of the petiole without further information (Cornelissen et al., 2003), with little clarity on the reasons for its inclusion or exclusion (Pérez‐Harguindeguy et al., 2013), or a total lack of discussion on the issue (Garnier et al., 2001). We suggest using the calculation of the leaf blade alone or without the petiole since both show the same tendencies and imply considerable reductions in measurement time.

## 5 CONCLUSIONS

This study provides evidence supporting the hypothesis that geological substrate acts as an environmental filter shaping leaf functional diversity in tropical dry forest communities. Overall, the limestone substrate was more benign for most edaphic variables, which resulted in a more functionally diverse community with a prevalence of trees with acquisitive leaf traits, unlike the phyllite substrate which was generally less benign (except for its higher total nitrogen content) and had a lower functional diversity and a prevalence of trees with more conservative leaf traits. This study contributes to the understanding of the role of the geological substrate as an environmental filter and its effect on the community leaf functional response, highlighting the importance of intraspecific functional variation.

## AUTHOR CONTRIBUTIONS

**Valentina Sandoval‐Granillo:** Conceptualization (equal); data curation (lead); formal analysis (lead); investigation (equal); methodology (lead); visualization (lead); writing – original draft (lead); writing – review and editing (equal). **Jorge A. Meave:** Conceptualization (equal); data curation (supporting); formal analysis (supporting); funding acquisition (lead); investigation (equal); methodology (supporting); project administration (lead); supervision (lead); validation (lead); visualization (supporting); writing – original draft (supporting); writing – review and editing (equal).

## CONFLICT OF INTEREST STATEMENT

The authors have no competing interests to declare.

## Supporting information


Data S1.

## Data Availability

The data that support the findings of this study are openly available in Dryad at https://doi.org/10.5061/dryad,zs7h44jf (Sandoval‐Granillo & Meave, 2023).

## APPENDIX 1

**TABLE A1:** Edaphic properties of soils derived from limestone and siliciclastic phyllite substrates in Nizanda, Oaxaca, Mexico.

Edaphic variable	Limestone mean ± SD (C.V., %)	Phyllite mean ± SD (C.V., %)
pH	6.42 ± 0.20 (3.12)	5.79 ± 0.34 (5.87)
Conductivity (μS)	567.43 ± 154.14 (27.16)	470.36 ± 131.46 (27.95)
Calcium (cmol(+)/kg)	48.63 ± 21.22 (43.64)	36.97 ± 14.88 (40.25)
Magnesium (cmol(+)/kg)	4.45 ± 1.93 (43.37)	4.62 ± 1.29 (27.92)
Sodium (cmol(+)/kg)	0.29 ± 0.06 (20.69)	0.31 ± 0.06 (19.35)
Potassium (cmol(+)/kg)	1.07 ± 0.41 (38.32)	0.73 ± 0.09 (12.33)
Available phosphorous (mg/kg)	13.6 ± 10.79 (79.34)	7.52 ± 2.21 (29.39)
Total nitrogen (mg/kg)	47.52 ± 30.98 (65.19)	68.07 ± 26.10 (38.34)
Nitrates (mg/kg)	39.48 ± 26.86 (68.03)	57.33 ± 33.11 (57.75)
Ammonium (mg/kg)	8.04 ± 3.62 (45.02)	10.73 ± 2.92 (27.21)
Organic matter (%)	19.40 ± 4.85 (23.19)	17.06 ± 4.76 (19.41)
Bulk density (g/cm^3^)	0.79 ± 0.1 (12.66)	1.04 ± 0.64 (61.54)
Particle density (g/cm^3^)	2.27 ± 0.19 (8.37)	2.16 ± 0.22 (10.19)
Porosity (%)	65.3 ± 2.29 (3.51)	61.7 ± 6.92 (11.22)
Gravimetric humidity (%)	4.03 ± 1.22 (30.27)	2.93 ± 0.88 (30.03)
Clay (%)	35.3 ± 5.55 (15.72)	31.5 ± 6.74 (21.40)
Silt (%)	18.7 ± 7.94 (42.46)	18.2 ± 3.47 (19.07)
Sand (%)	46.0 ± 9.43 (20.50)	50.3 ± 5.33 (10.60)
