# Supplementary material for: Leaf functional diversity and environmental filtering in a tropical dry forest: Comparison between two geological substrates
Source: Ecol Evol. 2023 Sep 5;13(9):e10491. doi: 10.1002/ece3.10491 (PMC10480066; doi:10.1002/ece3.10491)
Supplement: Supplementary file 1 — Data S1. [file ECE3-13-e10491-s001.docx]

**Leaf functional diversity and environmental filtering in a tropical dry forest: comparison between two geological substrates**

Valentina Sandoval-Granillo and Jorge A. Meave

Departamento de Ecología y Recursos Naturales, Facultad de Ciencias, Universidad Nacional Autónoma de México, Coyoacán, Ciudad de México, Mexico

Published in: *Ecology and Evolution*

# **Methods for soil analyses in the lab**

## **True density and bulk density**

Each one of the 14 samples from the 100 cm^3^ cylinder was emptied in a Petri dish and weighted in an analytical scale (Precisa, XT120A). Then these dishes were oven dried at 105 ºC one night or until the water from the substrate evaporated. Once dried they were weighed a second time. Bulk density was calculated by subtracting the dry soil weight from the fresh weight and dividing by the volume of the cylinder. The percentage of gravimetric humidity was calculated with the following formula:

$$\% gravimetric humidity= \frac{D_{w}-F_{w}}{F_{w}}\times100$$

With:

D_w_: soil dry weight

F_w_: Fresh weight

True density was determined with the pycnometer method, taking four different weights: empty pycnometer, sealed pycnometer filled to 1/3 with soil, sealed pycnometer full of soil and diluted to the mark with boiled water and last sealed pycnometer filled to the mark with water. This procedure was done for the 14 samples, each one with a duplicate, two controls and a duplicated stock solution. True density was calculated as:

$$TD=\frac{w2-w1}{\left( w2-w1 \right)-(w3-w4)}$$

With:

TD= true density

w1= empty sealed pycnometer

w2= pycnometer with 1/3 of soil

w3= sealed pycnometer filled to the mark

w4= pycnometer filled to the mark

With bulk and true densities, the pore volume was calculated with this formula:

$$PV=\frac{1-BD}{TD}\times100$$

With:

PV= pore volume

BD= bulk density

TD= true density

## **Interchangeable cation determination by extraction with ammonium acetate 1N pH 7**

For this analysis and the next we used mixed and oven dried samples. 2 g of soil were weighed for each sample in 50 ml tubes, including two reagent controls and the duplicated stock solution for each badge. 16 ml of ammonium acetate 1 N at pH 7 were added and mixed for 10 min. They were centrifuged at 2500 rpm for 5 min or until the supernatant was clear. This supernatant was filtered in a 50 ml tube. The extraction procedure was repeated twice (in the last wash, 18 ml were added instead of 16 to complete 50 ml). For the Ca^2+^ and Mg^2+^ determinations, we diluted with an automatic diluter (1:200), using a 0.5 % lanthanum solution. An atomic absorption spectrophotometer was used for the readings.

### *Calcium determination*

A calibration curve with 0, 1, 2, 4 and 6 mg/l of Ca was made: a volume of 1 ml of the 1000 mg/l calcium solution was weighed in a 15 ml tube with distilled water, gauging by weight to 10 ml of 0.5% lanthanum chloride which resulted in a 100 mg/l solution. From this, 0, 0.5, 1, 2 and 4 ml were weighted and put into 50 ml tubes. 50 ml of 0.5% lanthanum chloride were added. Then, ammonium acetate was added in an equivalent concentration required for the sample. For 1:100 dilutions or higher, a calibration curve with water and 0.5% lanthanum chloride was made.

### *Magnesium determination*

A curve with 0, 0.1, 0.3, 0.5, 0.7 and 1.0 mg/l of magnesium was constructed, for which a volume of 1 ml of the 1000 mg/l Mg solution was weighed and placed in a 15 ml tube with distilled water and adding water to the mark of the volumetric flask with 10 ml of 0.5% lanthanum chloride, resulting in a 100 mg/l solution. A volume of 1 ml of the 100 mg/l Mg solution was weighed in a 15 ml tube with distilled water, resulting in a 10 mg/l solution. Lanthanum chloride and ammonium acetate were added in the same manner as in the calcium curve. An atomic absorption spectrophotometer was used for the readings.

### *Potassium and sodium determination*

A calibration curve of de 0, 1, 2, 4, 6, 8 and 10 mg/l for Na^+^ and K^+^ was constructed, for which 0, 0.5, 1, 2, 3, 4 y 5 ml of the 100 mg/l solution of sodium and potassium were added into 50 ml tubes. A solution of ammonium acetate equivalent to the required concentration was added, also adding 0.1% cesium chloride. An emission spectrophotometer was used for the readings. These formulas were used to get the measurements in cmol(+)/kg units:

$$Ca mg/kg=\frac{\mathrm{reading}\left( mg/l \right)\times final volume \left( \mathrm{ml} \right)\times\mathrm{dillution}\left( \mathrm{ml} \right)}{soil weight \left( g \right)} Ca cmol(+)/kg=\frac{Ca (mg/kg)}{200.4}$$

$$Mg mg/kg=\frac{\mathrm{reading}\left( mg/l \right)\times final volume \left( \mathrm{ml} \right)\times\mathrm{dillution}\left( \mathrm{ml} \right)}{soil weight \left( g \right)} Mg cmol(+)/kg=\frac{Mg (mg/kg)}{121.5}$$

$$Na mg/kg=\frac{\mathrm{reading}\left( mg/l \right) \times final volume \left( \mathrm{ml} \right)\times\mathrm{dillution}\left( \mathrm{ml} \right)}{soil weight \left( g \right)} Na cmol(+)/kg=\frac{Na (mg/kg)}{230}$$

$$K mg/kg=\frac{\mathrm{reading}\left( mg/l \right) \times final volume \left( \mathrm{ml} \right)\times\mathrm{dillution}\left( \mathrm{ml} \right)}{soil weight\left( g \right)} K cmol(+)/kg=\frac{K (mg/kg)}{391}$$

## **Determination of available phosphorous (Bray-Kurtz method), using ascorbic acid as a reductant**

For each sample, 1 g of soil by duplicates was weighed in a 15 ml tube including the duplicated stock solution and two controls. 7 ml of extracting solution (NH4F at 0.03 M and HCl at 0.025 M was added. They were covered with parafilm and manually agitated for one minute. Immediately, the contents were filtered with filter paper (Whatman No. 40). If the filtered solution was not clear, it was filtered again through the same filter. The filtering process did not exceed 10 minutes. With an automatic pipette, 1 ml of each of the filter curve points of the samples, the stock solution and the two controls (each one in a different tube) were taken. To each tube, 2 ml of boric acid and 3 ml of reactive mixture (200 ml of distilled water, 50 ml of H_2_SO_4_ at 2.5 M, 15 ml of ammonium molibdate at 4 %, 30 ml of 1.75 % ascorbic acid solution and 5 ml de of 0.275 % potassium antimony tartrate) were added. The samples were homogenized and left to rest for at least an hour until the blue color was fully developed. Absorbance was measured in a Perkin Elmer (AAanalyst 800) spectrophotometer at 720 nm and the corresponding calculation to the readings was done, tracing a curve of mgP/kg and absorbency (The correlation coefficient value of the calibration curve must be at least 0.999 to be considered acceptable):

$$P \left( \frac{mg}{kg de suelo} \right)=\left( a-b \right)\times\left( \frac{ml of extracting solution}{soil weight g} \right)\times d=\left( a-b \right)\times\frac{7}{2} \times d$$

With:

P= phosphorous

a= mgP/l of the sample

b= mgP/l in the control

d= dilution factor

## **Determination of pH and electrical conductivity in aqueous soil extractions**

10 mg of each soil sample were weighted in 50 ml polypropylene tubes. 50 ml of distilled water were added, and the samples were shaken for 18 hours, leaving them to rest for one hour. They were centrifuged at 2000 rpm and the supernatant was read using the potentiometer. Then electrical conductivity was read with the aid of a conductivity meter.

##

## **Percentage of organic matter by calcination**

30 empty melting pots were weighted in the analytic scale and 5 g of soil were added, registering the weight of each one. They were introduced into a muffle at 480 ºC ± 10 ºC for 2 hours. The melting pots were taken out and into a desiccator with silica gel. An hour later they were weighted in an analytic scale. The formula used to calculate the percentage of organic matter was:

$$\% OM=\frac{W_{1}-W_{2}}{W_{2}-Mp}\times10$$

With:

W_1_ = soil weight previous to calcination

W_2_ = soil weight after calcination

Mp = empty melting pot weight

## **Soil texture determination by the Bouyoucos method (modified)**

30 g of each soil sample were weighted in 500ml jars, to which 100 ml of deionized water were added. Previous to the texture determination, the cementing agents in the samples must be destroyed.

*Organic matter destruction (only in samples with > 1% of OM)*

20 ml of 8% H_2_O_2_ were added to each sample before shaking with a glass rod, leaving them to rest until the reaction decreased. Then, the samples were placed in a water bath at a temperature not higher than 60 ^o^C and doses of 20 ml of 8% H_2_O_2_ were added until the following signals were observed:

The suspension no longer showed effervescence and the soil turned a darker color or the surface tension in the bubbles turned clear. This indicated the destruction of the organic matter. It is very important that the sample does not dry in the water bath, because its texture fractions could be fractured or lead to the formation of oxides.

*Sesquioxide destruction*

160 ml of 0.3 M sodium citrate and 40 ml of 1 M NaHCO_3_ were added to the soil samples after the destruction of organic matter (with a volume of around 50 ml). They were placed in a water bath at 75 ºC (without exceeding 80 ºC to avoid sulfur precipitation). The samples were stirred with a glass rod and 0.5 g of sodium dithionite after which they were left to rest in the bath for 20 minutes. After this time, the samples were placed in the centrifuge at 3000 rpm for 15 minutes (until the supernatant became clear). The entire procedure was repeated once more. In the case of samples not cementing in the centrifuge due to the saturation of the sodium exchange sites that have a dispersant effect, 10 ml of 1 M MgCl_2_ were added.

*Salt washing*

The samples were poured into Nalgene containers with distilled water and briefly stirred to centrifuge them at 3000 rpm for 15 minutes (until the supernatant became clear). The supernatant was decanted, and this was repeated until the electrical conductivity of each sample was lower than 100 µScm^-1^.

*Dispersion*

50 ml of 0.4 N (NaPO_3_)_6_ were added to the samples subjected to the pretreatments. These were shaken for an entire night. The next day they were placed in an ultrasound for 5 minutes. A control was made with 50 ml of (NaPO_3_)_6_ since it does not evaporate.

*Texture analysis*

Soil suspensions were placed in 1000 ml test tubes and diluted to the mark with distilled water. The temperature of each was measured and noted. The suspensions were stirred with a manual test tube agitator for 1 minute. Then they were left to rest for 40 seconds and the first hydrometer reading was taken, along with the temperature of the sample. This was left to rest for 2 hours and the second reading was taken. The temperature was taken and registered a third time.

The percentage of each soil textural phase were calculated as:

$$\% silt and clay= \frac{1^{st} corrected reading (40"\times100)}{soil g}-clay control$$

$$\% clay= \frac{2^{nd} corrected reading (120"\times100)}{g de suelo}$$

$$\% silt=\% silt and sand-\% clay$$

$$\% sand=100-(\% silt+\% clay)$$

The calculations were made and corrected by temperature and the textures of each sample were classified using the texture triangle.

## **Method for the nitrate and interchangeable ammonium in soil samples or sediments by potassium chloride extraction**

*Ammonium extraction*

10 g of dry soil per sample were weighted and sieved into Falcon tubes. 30 ml of 1M KCl were added. They were shaken in an orbital shaker for an hour and filtered through a Watman 42 paper filter. 3 ml of the extraction were taken and placed in glass test tubes with screw caps and the following solutions were added: 0.12 ml of phenol, 0.12 ml of sodium nitroprusside, 0.3 of oxidizing solution (made with alkaline reagent with 100 g of sodium citrate and 5 g of sodium hydroxide in 500 ml of deionized water with 25 ml of sodium hypochlorite). The samples were shaken in a vortex after each addition and kept 1 hour in the darkness. Two controls and the stock solution were prepared by pipetting extractive solution and adding the rest of the reagents.

Calibration curve was made preparing standards between 0.1 and 100 mg NH_4_/l in a volume of 10 ml using a stock solution of 1000 mg NH_4_/l and 1 M KCl as a dissolution medium. Absorbance was read with a spectrophotometer at 640 nm. All of the materials in contact with the solutions were washed with acidulated HCl water at a 10% concentration.

*Nitrate extraction*

0.5 ml of each one of the extractions were mixed with 1 ml of salicylic acid and stirred with the aid of a vortex. 10 ml of 4 N NaOH were added and the solution was mixed once again. The samples were left to cool at room temperature. Two controls and a stock solution were made by pipetting extractive solution and adding the salicylic acid and the NaOH. A calibration curve was made preparing standards between 0.5 and 30 mg N-NO_3_/l in a volume of 10 ml, using the stock solution 100 mg N-NO_3_/l and 1 M KCl as a dissolution medium. Absorbance was read at 410 nm. All of the material in contact with the solutions was washed with acidulated HCl water at a 10% concentration.

**References**

Bray, R.H. and Kurtz, L.T., 1945. Determination of total, organic, and available forms of phosphorus in soils. *Soil Science*, *59*(1), pp. 39-46. http://dx.doi.org/10.1097/00010694-194501000-00006

Bouyoucos, G.J., 1962. Hydrometer method improved for making particle size analyses of soils 1. *Agronomy Journal*, *54*(5), pp. 464-465.

https://doi.org/10.2134/agronj1962.00021962005400050028x

Mylavarapu, R., Bergeron, J. and Wilkinson, N. 1993. Soil pH and electrical conductivity: a county extension soil laboratory manual (downloaded on March 17, 2020 from: https://edis.ifas.ufl.edu/pdffiles/SS/SS11800.pdf.
